# Validation of AAC-11-Derived Peptide Anti-Tumor Activity in a Single Graft Sézary Patient-Derived Xenograft Mouse Model

**DOI:** 10.3390/cells11192933

**Published:** 2022-09-20

**Authors:** Justine Habault, Nicolas Thonnart, Caroline Ram-Wolff, Martine Bagot, Armand Bensussan, Jean-Luc Poyet, Anne Marie-Cardine

**Affiliations:** 1 INSERM U976 Team 1, Onco-Dermatology and Therapies, 75010 Paris, France; 2 Saint Louis Research Institute, Université Paris Cité, 75010 Paris, France; 3 Department of Dermatology, Saint Louis Hospital, AP-HP, 75010 Paris, France

**Keywords:** Sézary syndrome, Sézary patient-derived xenograft murine model, AAC-11, anti-tumor peptide, cell-penetrating peptide

## Abstract

Sézary syndrome (SS) is an aggressive cutaneous T cell lymphoma with poor prognosis mainly characterized by the expansion of a tumor CD4^+^ T cell clone in both skin and blood. So far, the development of new therapeutic strategies has been hindered by a lack of reproducible in vivo models closely reflecting patients’ clinical features. We developed an SS murine model consisting of the intravenous injection of Sézary patients’ PBMC, together with a mixture of interleukins, in NOD-SCID-gamma mice. Thirty-four to fifty days after injection, mice showed skin disorders similar to that observed in patients, with the detection of epidermis thickening and dermal tumor T cell infiltrates. Although experimental variability was observed, Sézary cells could be tracked in the blood stream, confirming that our model could efficiently exhibit both skin and blood involvement. Using this model, we evaluated the therapeutic potential of RT39, a cell-penetrating peptide derived from the survival protein anti-apoptosis clone 11 (AAC-11), that we previously characterized as specifically inducing apoptosis of Sézary patients’ malignant clone ex vivo. Systemic administration of RT39 led to cutaneous tumor T cells depletion, demonstrating efficient malignant cells’ targeting and a favorable safety profile. These preclinical data confirmed that RT39 might be an innovative therapeutic tool for Sézary syndrome.

## 1. Introduction

Cutaneous T cell lymphomas (CTCL) are a heterogenous class of non-Hodgkin’s lymphomas characterized by the clonal proliferation of neoplastic T cells originating from the skin. The two main forms are mycosis fungoides (MF), where the tumor T cells remain confined to the skin, and Sézary syndrome (SS), where tumor cells show cutaneous but also blood location, this leukemic presentation leading to severe immunosuppression at advanced stages and to consequently poor prognosis. Although currently available treatments allow control of early stages MF, there is still no cure for advanced stages MF and SS [1]. In this regard, anti-cancer peptides may represent a suitable alternative as their mode-of-actions do not rely on the integrity of the immune system [2].

AAC-11 (anti-apoptosis clone 11, also called Api5 or FIF), is an anti-apoptotic protein whose expression prevents apoptosis following growth factor deficiency [3]. Experimental data indicated that AAC-11, which is overexpressed in a number of cancer tissues and associated with bad prognosis, is involved in the regulation of cancer cells metastasis and associated with aggressive tumor behavior through an upregulation of matrix metalloproteinases involving the ERK/AP-1 pathway [4,5,6]. It has also been reported that decreased expression of various tumor suppressor microRNAs in several cancers was correlated with AAC-11 upregulation, which might be linked to tumor progression [7,8,9,10]. Furthermore, we and others showed that AAC-11 gene silencing remarkably decreased chemoresistance, whereas its expression interfered with drug-induced apoptosis [11,12,13]. Collectively, these observations indicate that AAC-11 constitutes an attractive target in cancer therapy given its role in both cancer progression and therapy resistance.

AAC-11 functions as a scaffold for several apoptosis-related proteins, such as FGF2, ALC1 and Acinus (see [14] for review). This scaffolding function appears to play a critical role in cancer cells survival. AAC-11 contains multiple protein–protein interaction modules including a heptad leucine repeat region [15]. Interestingly, inactivating mutations within this region prevents the AAC-11 interaction with Acinus and abrogates its anti-apoptotic and pro-metastatic effects, indicating a critical role for this domain in AAC-11 biological functions [14]. We recently reported the generation of a cell-penetrating peptide (hereafter called RT39), encompassing the minimal stretch of amino acids of AAC-11 heptad leucine repeat region required for interaction with its binding partners. We previously showed that this peptide was able to specifically target and induce depletion of Sézary patients’ circulating malignant T cell clone ex vivo while sparing non-malignant immune cells [16].

Although numerous CTCL mouse models were already described, they often presented features that do not correspond to the spectra of patients’ disease progression or genetic background (e.g., use of non-CTCL or CTCL cell lines rather than patients’ derived cells, development of tumor at the point of injection (e.g., subcutaneous or intra-hepatic) with no dissemination of tumor cells in the skin, occurrence of multi-organ metastases or need for complex genetically engineered models) [17,18,19,20,21,22,23,24,25,26,27,28]. More recently, new patient-derived xenograft (PDX) models were reported allowing efficient expansion of patients-derived malignant cells both in vitro and in vivo and ultimately anti-tumor agents testing [29,30]. However, cutaneous involvement was not obtained after the first engraftment but required a secondary xenograft of tumor cells isolated and amplified from the first generated animals [29,30].

So far, RT39 anti-tumor activity has been estimated on a classical Sézary cell line (HUT78) subcutaneous xenograft mouse model [16]. We here sought to confirm its efficiency on a Sézary PDX mouse model exhibiting clinical features as close as possible to the one encountered in patients.

## 2. Materials and Methods

### 2.1. Peptides Synthesis

Peptides were synthesized by Proteogenix (Strasbourg, France) and were >95% pure, as determined by HPLC and mass spectrographic analysis. Sequences are: RT39: RQIKIWFQNRRMKWKKLQYFARGLQVYIRQLRLALQGGKT; RT39M: RQIKIWFQNRRMKWKKLQYFAAGLQVYIRQLRLALQGGKT

### 2.2. Patients

The study was approved by the institutional ethics committee (Saint Louis Hospital, Paris) and blood was collected after obtaining patients’ written informed consent. Peripheral blood mononuclear cells (PBMC) were isolated from heparinized blood samples by gradient centrifugation over lymphocytes separation medium (Eurobio, France). All selected patients presented high tumor burden (B2) and skin involvement (T2b or T4) at the time of blood sampling.

### 2.3. Flow Cytometry

Flow cytometry determination of the tumor clone TCR-Vβ rearrangement was performed using the TCR-Vβ repertoire kit from Beckman Coulter. Lymphocyte content was determined by immunolabeling with a mix of anti-human-CD3, -TCR-Vb, -CD4, -CD8, -KIR3DL2, -CD45 and -CD56 antibodies or their corresponding control isotypes. After acquisition on a CytoFlex cytometer (Beckman Coulter), data analysis was performed using FlowJo software (TreeStar Inc., San Carlos, CA, USA).

### 2.4. Generation of PDX Mice and Peptide Treatment

Eight-week-old female NOD-SCID-gamma (NSG) mice were injected intravenously with freshly isolated PBMC supplemented with IL2 and IL7 (2 × 10^7^ cells/400 UI IL2/60 ng IL7 per mouse). Injection of interleukins was continued once a week during the entire course of the experiment. Mice were monitored daily for potential weight loss, occurrence of abnormal posture or behavior and cutaneous changes/deterioration. Blood sampling was performed once a week for circulating T cells monitoring by flow cytometry.

For peptide treatment (Experiment 4), mice were randomized into 3 groups (*n* = 5/group) when cutaneous involvement was reached (Day 55). RT39 or RT39M (5 mg/kg in normal saline) were injected intra-peritoneally every two days. Mice were euthanized when human or experimental end point criteria were met (Day 84 for untreated and RT39M groups; Day 120 for RT39 group).

### 2.5. Immunohistochemistry (IHC) and Immunofluorescence (IF)

Skin biopsies were collected at lesion sites before peptide treatment (Experiment 4) or/and at sacrifice/end of treatment (EOT) (Experiments 1–4). In Experiment 4, EOT biopsy was performed 2–3 mm apart from the first biopsy on a non-overlapping location. Samples were divided into two pieces and included either in paraffin (IHC) or OCT (IF). Six-micrometer thick sections were prepared and processed for IHC or IF staining with anti-CD3 (polyclonal rabbit IgG) and -KIR3DL2 (clone 12B11; muIgG1; kindly provided by Innate Pharma, Marseille, France) antibodies, according to standard protocols.

After CD3 staining, epidermis thickness measurement (7 points of measurement per biopsy) and CD3^+^ T cells enumeration were performed blindly on skin sections corresponding to 3 mice in each group. Similar analysis was realized on control/not engrafted mice (*n* = 3).

## 3. Results

### 3.1. Generation and Characterization of a Novel Single Graft Sézary PDX Mouse Model

In an attempt to obtain a CTCL mouse model that encompasses cutaneous and/or blood involvement readily after the first engraftment, we adopted a PDX-based approach with Sézary patients’ PBMC as the source of tumor cells and NOD-SCID-gamma (NSG) mice as recipients. To limit the occurrence of graft versus host disease (GVHD) reaction, we selected patients presenting a high circulating tumor burden (B2 stage) for PBMC engraftment, with malignant cells (identified by flow cytometry on the basis of their TCR-Vβ rearrangement) representing over 85% of the total CD4^+^ T cell population. The main clinical and cellular characteristics of the patients whose PBMC were used to establish the PDX mice are described in Table 1. More importantly we added a mixture of IL2 and IL7 at the time of cells’ injection and then once a week during the entire course of the experiment. Indeed, abnormal expression of CD25 and/or IL7-receptor was often found associated to CTCL, and these cytokines previously allowed us to select and generate T cell lines from SS patients’ circulating malignant T cell clones [31,32,33,34].

Cells and cytokines were injected intravenously (i.v.). Three pilot experiments were first conducted, each one using PBMC from one SS patient (Experiments 1–3; *n* = 3/experiment). Out of nine engrafted mice, eight showed skin disorders 34 to 50 days post-engraftment that mimic CTCL presentation (erythrodermic appearance (Experiment 1) or patch/plaque evolving toward transformed-like appearance (Experiments 2 and 3; Supplementary Figure S1A)). One animal developed early GVHD symptoms (see experiments summary given in Supplementary Table S1). Of note, no apparent sign of tumor evasion toward visceral organs was detectable (Supplementary Figure S1B). However, spleen enlargement was observed (*n* = 7/8; Supplementary Figure S1C), with the presence of T cell infiltrates containing scarce malignant T cells detected through the expression of KIR3DL2 receptor (previously identified as a reliable malignant T cell marker [35,36]) (Supplementary Figure S1D). Finally, variations in the % of circulating CD4^+^ T cells, as well as in the % of circulating tumor cells, were observed from one animal to another in the same experimental group, but also from one experiment to another (summary of mice skin and blood main features given in Supplementary Table S1).

**Figure 1:**
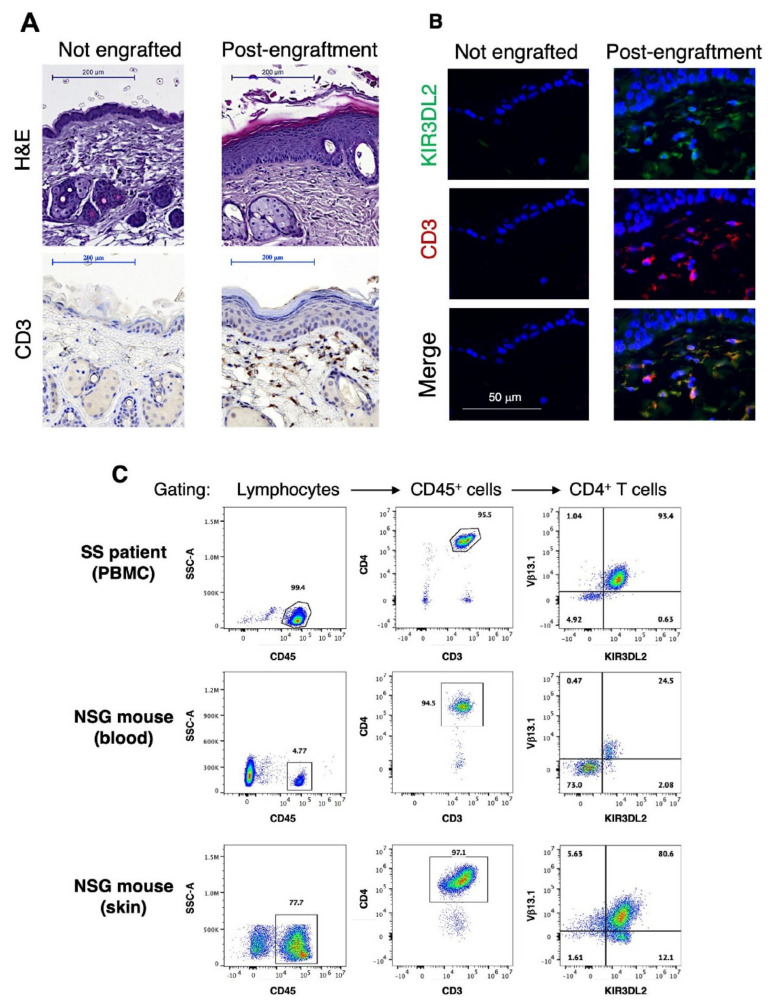
Main skin and blood features of the SS PDX mouse model. (**A**) Hematoxylin–eosin (upper panel) or CD3 (lower panel) staining was performed on skin sections from control (not engrafted) mouse (left) and 80 days after engraftment with Sézary patient’s PBMC (right). Scale bar = 200 µm. (**B**) Skin sections from control (left panels) and engrafted (right panels) mice were subjected to immunofluorescence labeling using anti-KIR3DL2 (clone 12B11) and anti-human CD3 antibodies plus AlexaFluor488 (green)- and AlexaFluor594 (red)-coupled secondary reagents. Slices were mounted using a Dapi-containing mounting medium (blue). Scale bar = 50 µm. (**C**) Flow cytometric analysis of the tumor T cell clone following mice engraftment. Sézary patient PBMC (upper panels) and lymphocytes recovered either in the blood (middle panels) or after collagenase extraction of a skin fragment (bottom panels) of a representative engrafted mouse were labeled with a mix of anti-human CD45, CD3, CD4, KIR3DL2 and TCR-Vβ antibodies. Following successive gating of lymphocytes, CD45^+^ cells and CD4^+^ T cells, the patient original malignant T cell clone was identified through its TCR-Vβ13.1 rearrangement and expression of KIR3DL2 tumor marker.

Further analysis of skin sections by immunohistochemistry clearly revealed a tremendous modification of the skin architecture of the engrafted mice compared to untreated animals, with the detection of multilayers of keratinocytes, characteristic of epidermis thickening, and of dermal CD3^+^ T cell infiltrates (Figure 1A), two main features of CTCL. In addition, immunostaining analysis allowing detection of human CD3 and KIR3DL2 tumor marker confirmed the presence of double positive cells in the dermis (Figure 1B). Following lymphocytes extraction from engrafted mice skin sections and flow cytometry analysis, the majority of cutaneous CD4^+^ T cells was found to share the same TCR-Vβ rearrangement and KIR3DL2 expression as the patient’s original circulating malignant T cell clone (Figure 1C). Finally, clonal KIR3DL2^+^ CD4^+^ T cells could also be detected in the mice blood stream during the course of the experiment (e.g., in Figure 1C).

Taken together, our data showed that our SS PDX mouse model recapitulates the main features of SS, namely a dermal infiltration by malignant T cells leading to cutaneous disorders associated to blood tumor dissemination, while no major life-threatening events were detected.

### 3.2. RT39 Treatment Promotes Depletion of the Tumor T Cell Clone in SS PDX Mouse Dermis

We previously demonstrated that RT39, but not its inactive point mutated form RT39M, exhibited a selective cytotoxicity toward Sézary patients’ primary tumor CD4^+^ cells ex vivo, while sparring non-malignant lymphocytes [16]. Since our experimental design led to the generation of mice presenting blood and cutaneous involvement with no occurrence of life-threatening adverse effects requiring early mice sacrifice, we aimed to validate RT39 anti-tumor activity in this PDX mouse model (Experiment 4).

**Figure 2:**
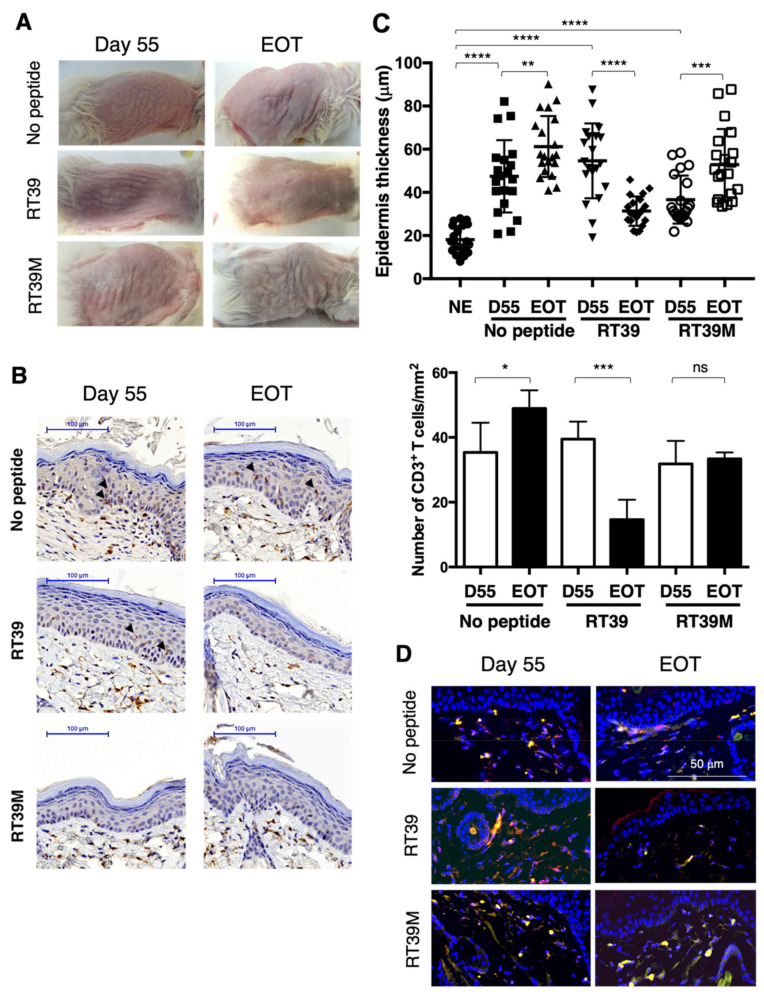
In vivo validation of RT39 peptide anti-tumor efficiency. After engraftment and appearance of cutaneous manifestations (55 days post-injection), mice were randomly separated in 3 groups (*n* = 5 each) and either left untreated (no peptide) or treated with RT39 or RT39M peptide. Skin biopsies were collected before (Day 55) and at the end of treatment (EOT). (**A**) Visual evaluation of cutaneous disorders before and after peptide treatment. Shown are pictures of one representative mouse from each group. (**B**) Immunohistochemical analysis of skin sections. Labeling was performed using an anti-human CD3 mAb, allowing observation of the epidermis thickness and of epidermal (arrowheads) and dermal T cell infiltrates. Shown are representative data obtained on one representative mouse from each group. (**C**) After CD3 staining, epidermis thickness was measured (7 points of measurement; upper panel) and CD3^+^ T cells were enumerated (bottom panel) on skin sections corresponding to 3 mice in each group. Similar analysis was realized on control/not engrafted mice (NE; *n* = 3). Statistical analysis was performed using a Mann–Whitney t test. * *p* < 0.05, ** *p* < 0.01, *** *p* < 0.001, **** *p* < 0.0001, ns: not significant. (**D**) Analysis of the presence of the malignant T cell clone by immunofluorescence using human CD3 and KIR3DL2 as tumor T cell marker. Shown are representative data obtained on one mouse from each group.

After engraftment and development of cutaneous disorders (Day 55), mice were randomized in three groups and either left untreated or intra-peritoneally injected with RT39 peptide or its inactive mutated version devoid of cytotoxic activity RT39M (5 mg/kg every two days) [37]. At the end of treatment, RT39 anti-tumor efficiency was readily detectable through visual examination of the skin, such cutaneous improvement not being observed in untreated or RT39M-treated mice (Figure 2A). RT39-mediated depletion of cutaneous malignant T cells was further evaluated by performing IHC analysis on skin biopsies sampled before and after treatment. As shown in Figure 2B, the RT39-treated group showed an epidermis structure that tends toward normal. Quantification of the epidermis thickness and of the number of infiltrating T cells confirmed skin improvement and dermal T cell depletion upon RT39 treatment (Figure 2C). Note that in this experiment, epidermal T cell infiltrates were detectable before but not after RT39 treatment (Figure 2A, arrowheads). Finally, IF labeling allowed validation of tumor T cells’ clearance with the disappearance of infiltrating KIR3DL2^+^ T cells. In contrast, untreated and RT39M-treated mice experienced no improvement nor a worthening of their epidermis thickness with no clearance of the infiltrating CD3^+^ T cells (Figure 2B,C) or KIR3DL2^+^ malignant T cells (Figure 2D).

Unfortunately, blood involvement was not achieved in any of the groups. Indeed, less than 2% of circulating T cells were detected in all mice post-engraftment and, thereafter, at intermediate time points until EOT, preventing evaluation of the peptide efficacy on the blood tumor burden (see experiment summary in Supplementary Table S1).

Nevertheless, our data demonstrated that RT39 displays potent and specific anti-tumor efficiency against the dermal infiltrating malignant T cell clone, consequently leading to a significant reduction in the cutaneous deterioration.

## 4. Discussion

Peptide-based therapies present a number of advantages over traditional compounds such as small molecules as, beside their increased specificity, peptides allow targeting of protein–protein interactions, thereby providing an array of new therapeutic targets and clinical applications. We have recently developed a cell-penetrating peptide, RT39, that can disrupt the interactions between the survival factor AAC-11 and its binding partners, resulting in Sézary patients’ primary malignant T cells’ death [16]. To date, the lack of representative and easy to generate preclinical animal models has hampered the validation of new therapeutic alternatives for the treatment of Sézary syndrome [37]. We here describe the successful generation of a Sézary PDX mouse model that, unlike previously reported CTCL models, displays cutaneous involvement after a unique injection of patients’ cells. Our model essentially relies on i.v. engraftment of PBMC isolated from patients with high tumor burden associated to a weekly supply of IL2 and IL7 cytokines.

Skin disorders, associated with the presence of dermal T cell infiltrates and more occasionally to epidermal infiltration, were observed in all sets of experiments. In contrast, detection of circulating malignant cells showed higher inter- and intra-experiment variation. However, it should be noted that the engrafted mice had to be sacrificed no later than 3 months post-engraftment because of heavy skin deterioration, caused by the disease-related skin disorders, leading to high levels of animal discomfort and/or post-scraping infection. Because malignant cells’ migration from skin to blood is an event symptomatic of advanced stages of Sézary syndrome, one has to consider that it might not be systematically achieved in mice in case of high cutaneous damage. Nevertheless, the engrafted mice still presented a lifetime allowing long-term anti-tumor assays. Therefore, our murine model should provide an interesting preclinical platform for the in vivo screening of novel therapeutic agents.

Taking advantage of our mouse model characteristics, we provide a proof-of-concept regarding the use of RT39 anti-tumor peptide as a therapeutic tool in the context of CTCL. Indeed, upon systemic administration, RT39 showed profound anti-cancer effects when used as a single agent, as demonstrated by the efficient depletion of the infiltrating malignant T cell clone and consequent decrease in skin disorders. In our experimental setting, RT39 dosage was rather low (5 mg/kg) with an every other day schedule. Using this routine, RT39 appeared well-tolerated, with no apparent toxicity for the animals that can be maintained up to 4 months upon treatment. In addition, RT39 demonstrated efficient biological activity for targeting and depleting malignant T cells in the skin compartment. Unfortunately, an absence of blood involvement in the engrafted mice prevented evaluation of RT39 efficiency on the circulating malignant burden. However, our previous data demonstrated a specific depletion of malignant T cells, and not of the other immune cell compartments, by RT39 when added ex vivo to Sézary patients’ PBMC [16], favoring the idea that malignant clearance could also occur in vivo.

It is well-established that SS patients experience immunodeficiency and opportunistic infections upon disease progression [38]. By pre-selecting, as PBMC donors, patients presenting a high circulating tumor burden, and, therefore, strongly impaired NK and CD8^+^ T cell repertoires, one can reasonably think that their immunodeficient profile was conserved by the engrafted mice. We recently reported that RT39-mediated malignant cell depletion relied on its interaction with constitutively activated and plasma-membrane-located PAK1 leading to membranolysis [37]. Our data, therefore, confirmed that RT39 mode-of-action does not rely on immune integrity and argue for a therapeutic window for RT39 use especially for late-stage Sézary patients whose prognosis remains very poor. Moreover, considering that the skin is a key target compartment for CTCL, one could hypothesize that, beside Sézary syndrome, other cutaneous lymphomas, such as MF (where tumor cells remain essentially confined to the skin), might also benefit from a RT39-based therapy.

In conclusion, thanks to the generation of a single graft Sézary PDX mouse model presenting cutaneous disorders but no severe adverse effects requiring premature mice sacrifice, our results constitute preclinical data confirming that RT39 peptide might be an innovative therapeutic tool for Sézary syndrome.

## Figures and Tables

**Table 1:** Sézary patients’ main features and lymphocytes content prior to engraftment *.

Experiment n°	Patient			% of Cell Subset in PBMC			% of Tumor Cells in CD4^+^ T Cells **
	n°	Sex/Age	TNMB Classification	CD4^+^ T Cells	CD8^+^ T Cells	NK Cells	
**1**	1	F/62	T2bN0M0B2	74.1	1.7	<1%	93.7
**2**	2	M/65	T4N3M0B2	66.5	5.2	<1%	86.3
**3**	3	F/89	T2bNXM0B2	92.3	1.9	1.4	92.1
**4**	4	M/82	T4NXM0B2	97.1	<1%	n.d	99.7

* Determined by flow cytometry using a combination of anti-CD45, -CD3, -TCR-Vβ, -CD4, -CD8, -CD56 and KIR3DL2 antibodies; n.d: not detected. ** According to TCR-Vb rearrangement.

## Data Availability

Not applicable.

## Supplementary Materials

The following supporting information can be downloaded at: https://www.mdpi.com/article/10.3390/cells11192933/s1, Figure S1: Main organs characteristics encountered in Sézary PDX mice; Table S1: Main cutaneous and blood features of the engrafted mice.

## Abbreviations

SS: Sézary syndrome; MF, mycosis fungoides; CTCL, cutaneous T cell lymphoma; AAC-11, anti-apoptosis clone-11; PBMC, peripheral blood mononuclear cells; PDX, patient-derived xenograft.
