# Supplementary material for: Validation of AAC-11-Derived Peptide Anti-Tumor Activity in a Single Graft Sézary Patient-Derived Xenograft Mouse Model
Source: Cells. 2022 Sep 20;11(19):2933. doi: 10.3390/cells11192933 (PMC9564267; doi:10.3390/cells11192933)
Supplement: Supplementary file 1 [file cells-11-02933-s001.zip › Figure S1.pdf]

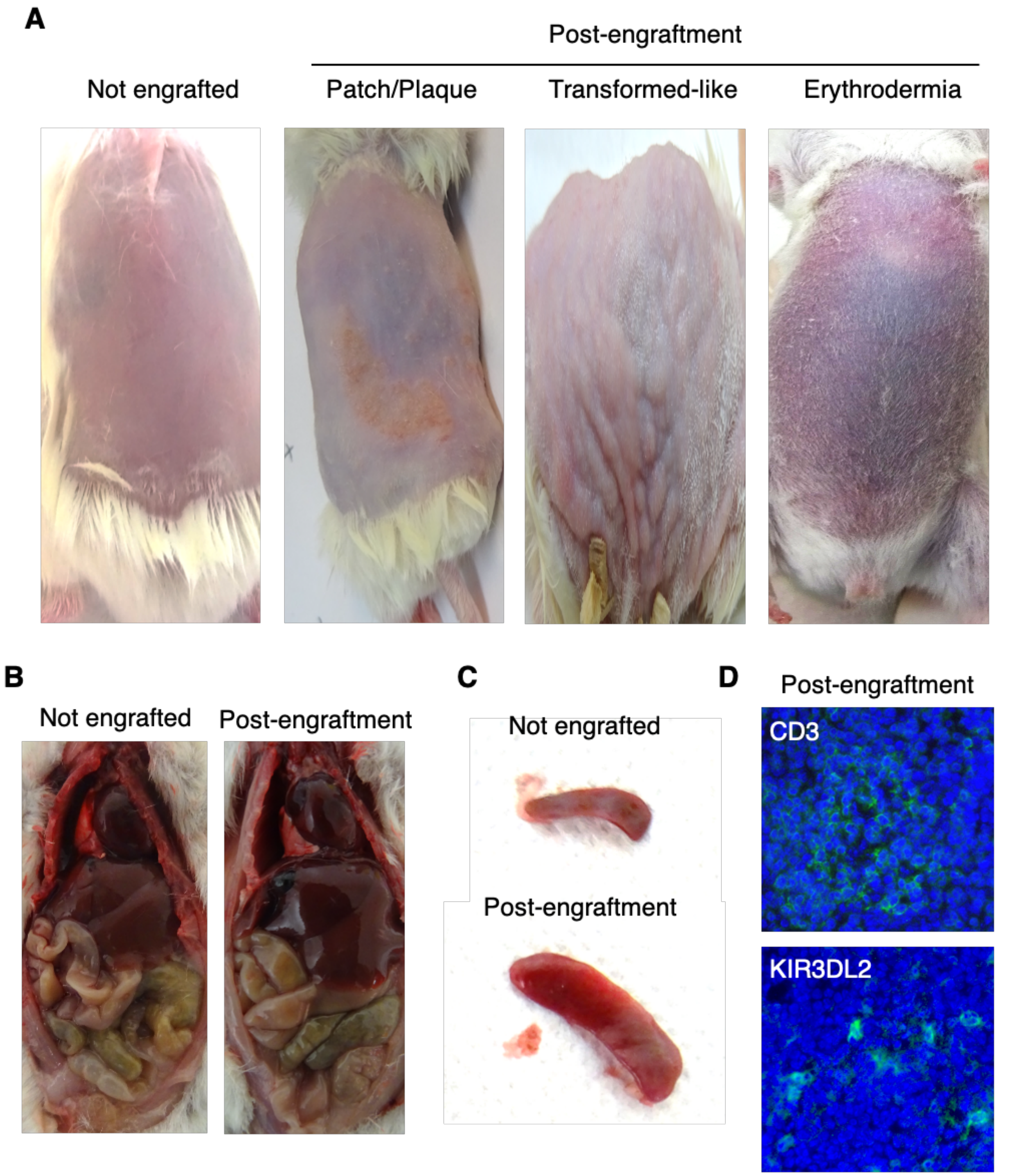

Figure S1: Main organs characteristics encountered in Sézary PDX mice. **(A)** Visual evaluation of the different types of cutaneous disorders observed post-engraftment. **(B)** Absence of noticeable tumor development in visceral organs **(C)** Spleen enlargement (n=7/8). **(D)** Immunofluorescence analysis of a representative enlarged spleen showing the presence of numerous $CD3^+$ T cells but only few malignant $KIR3DL2^+$ cells.
