# Supplementary material for: Validation of AAC-11-Derived Peptide Anti-Tumor Activity in a Single Graft Sézary Patient-Derived Xenograft Mouse Model
Source: Cells. 2022 Sep 20;11(19):2933. doi: 10.3390/cells11192933 (PMC9564267; doi:10.3390/cells11192933)
Supplement: Supplementary file 1 [file cells-11-02933-s001.zip › Table S1.pdf]

Table S1: Main cutaneous and blood features of the engrafted mice

Experiment n°	n	End Point (Day post-engraftment)	Adverse effects leading to sacrifice	Cutaneous manifestations			Blood involvement at end point*		
			n (Cause; Day post-engraftment)	n	Appearance (Day post-engraftment)	Type	n	% $CD4^+$ / T cells	% $V\beta^+$ $KIR3DL2^+$/ $CD4^+$ T cells
1	3	63	1/3 (GVHD; Day 38)	2/3	Range: 45-50	Erythrodermia	1/2	98.3	25.1
2	3	60	none	3/3	Range: 34-49	Patch/Plaque evolving towards transformed-like	3/3	Mean±SD: 91.7±6.8 Range: 84.0-96.6	Mean±SD: 11.9±14.5 Range: 3.1-28.7
3	3	70	1/3 (infection due to scratching; Day 53)	2/3	Range: 35-40	Patch/Plaque evolving towards transformed-like	3/3	Mean±SD: 67.1±30.4 Range: 34.6-94.7	Mean±SD: 36.8±53.1 Range: 3.2-98.8
4	15	NT/RT39M: 84 RT39:120	none	15/15	Range: 32-38	Transformed-like	less than 2% of circulating T cells at experiment end point for all groups		

* the % of $CD4^+$ T cells among circulating T cells (identified as $CD45^+$ $CD3^+$ cells), and of malignant TCR-$V\beta^+$ $KIR3DL2^+$ cells within $CD4^+$ T cells, were determined by flow cytometry on mice blood using anti-human-CD45, -CD3, -CD4, -KIR3DL2 and -TCR-Vβ mAbs.
